# Risk factors of non-union in intramedullary stabilized diaphyseal long bone fractures: identifying the role of fracture stabilization strategies and concomitant injuries

**DOI:** 10.1007/s00068-020-01335-y

**Published:** 2020-03-05

**Authors:** Martijn Hofman, Hagen Andruszkow, Frans L. Heyer, Philipp Kobbe, Frank Hildebrand, Martijn Poeze

**Affiliations:** 1 Department of Orthopedic Trauma and Reconstructive Surgery, University Hospital RWTH Aachen, Pauwelsstraße 30, 52074 Aachen, Germany; 2 Division of Traumasurgery,, Department of Surgery, Maastricht University Medical Center, P. Debyelaan 25, 6229 HX Maastricht, The Netherlands

**Keywords:** Non-union, Treatment strategy, DCO, ETC, Chest injury, TBI

## Abstract

**Purpose:**

Concomitant chest injury is known to negatively affect bone metabolism and fracture healing, whereas traumatic brain injury (TBI) appears to have positive effects on bone metabolism. Osteogenesis can also be influenced by the timing of fracture stabilization. We aimed to identify how chest injuries, TBI and fracture stabilization strategy influences the incidence of non-union.

**Methods:**

Patients with long bone fractures of the lower extremities who had been treated between 2004 and 2014 were retrospectively analysed. Non-union was defined as fracture healing not occurring in the expected time period and in which neither progression of healing nor successful union is expected without intervention. Diverse clinical and radiological parameters were statistically analysed using the Statistical Package for the Social Sciences (SPSS).

**Results:**

The total number of operations before consolidation was an independent predictor (odds ratio [OR] = 6.416, *p* < 0.001) for the development of non-union in patients with long bone fractures. More specifically, patients treated according to the damage control orthopaedics (DCO) principle had a significantly higher risk of developing a non-union than patients treated according to the early total care (ETC) principle (OR = 7.878, *p* = 0.005). Concomitant chest injury and TBI could not be identified as influencing factors for non-union development.

**Conclusion:**

Our results indicate that the number of operations performed in patients with long bone fractures should be kept as low as possible and that the indication for and the timing of DCO treatment should be meticulously noted to minimize the risk of non-union development.

## Introduction

Fracture healing depends on the interactions of many biomechanical and biological factors [1]. Disturbances in this process might result in non-union with an overall incidence of 1.5–10%, increasing up to 40% in case of open fractures. In particular, non-unions of the lower extremities have been identified to significantly impair the post-traumatic quality of life [2], and have been associated with high direct and indirect costs [3, 4].

The risk factors for non-unions might either arise from injury characteristics, patient-specific factors, or from parameter associated with surgical fracture stabilization [5, 6]. Interactions between local and systemic inflammatory responses have been considered as the potential reasons for delayed fracture healing in chest trauma [6]. In contrast, TBI seems to be positively correlated with osteogenesis [7–9]. However, this association has not been found in all studies [10, 11]. Furthermore, potential pathophysiological mechanisms for TBI-related impact on osteogenesis seem to be multifactorial (humoral, hormonal and cellular) and are far from clear [7, 12, 13].

Because of the enormous incapacitating effect of non-unions on the physical and mental health of patients, knowledge about the relevance of potential risk factors is of utmost importance. As the impact of several patient—(e.g. substance abuse, long-term use of steroids) and injury-specific (e.g. Gustilo type III open fracture) factors has already been well described [5], we focused on the influence of chest injury, TBI and fracture stabilization strategies in patients with long bone fractures of the lower extremities. Identifying the risk and predictive factors of non-union can help further develop prophylactic and therapeutic strategies for its treatment.

## Materials and methods

### Study design and exclusion criteria

We retrospectively analysed patients with diaphyseal femoral or tibial fractures who had been admitted and treated definitively with a reamed intramedullary locking nail at the Department of Orthopaedic Trauma and Reconstructive Surgery, University Hospital RWTH Aachen (Germany), or the Department of Traumasurgery, Maastricht University Medical Centre (The Netherlands) between 2004 and 2014. Clinical records and X-rays were retrieved for analysis. The patients’ clinical course was followed until the last outpatient appointment. Patients who developed a non-union were placed in the NU group and those with normal fracture healing were placed in the control group. Non-union was defined as fracture healing not occurring in the expected time period and in which neither progression of healing nor successful union is expected without intervention.

To restrict the number of previously described factors influencing non-union development and to focus on chest injury, TBI and fracture stabilization strategy (ETC vs. DCO) as the influencing factors, we applied the following exclusion criteria: 17 < age (years) < 80, substance abuse (alcohol, tobacco, drugs), morbid obesity (BMI > 30), mental disability, pregnancy, long-term use of steroids, bisphosphonates or thyroxin, lost to follow-up < 1 year after trauma, Severe soft tissue damage (Gustilo and Anderson > 2), Comminuted fracture, bone defect > 3 cm, Pathological fractures, fractures of an adjacent joint, bilateral fractures, definitive treatment other than reamed intramedullary locking nail and primary definitive treatment elsewhere.

### Treatment algorithm

All the patients were managed according to the principles of Advanced Trauma Life Support^®^ (ATLS^®^) and the S3 guidelines on the treatment of patients with severe injuries. For fracture treatment, patients underwent ETC with an antegrade intramedullary reamed locking nail, and if necessary, DCO at the earliest possible opportunity with an external fixator, which was subsequently converted to definitive osteosynthesis as soon as it was tolerated by the patient’s clinical condition. Intravenous antibiotic prophylaxis was given in closed fractures as a single dose and for 3 days in open fractures. As soon as their clinical state allowed it, patients were mobilized with partial and consecutive increase to full weight-bearing, according to the fracture type. After discharge, the patients were seen in the outpatient clinic at 2, 6, 12, 26 and 52 weeks postoperatively. If union was not achieved at that time point, further controls took place until union was achieved or a revision was indicated.

### General health status and injury severity

The general health status of the patients was estimated according to the American College of Anaesthesiologists (ASA) classification system and the Charlson comorbidity index (CCI) [14], which calculates an estimated relative risk of death based on the patient’s age, cardiopulmonary and cerebrovascular condition, the presence of metabolic, gastrointestinal and infectious diseases as well as malignancies. Overall, injury severity was determined with the 2005 revised edition of the Abbreviated Injury Scale (AIS) and summarized to the Injury Severity Score (ISS) [15].

### Classification of chest injury

Concomitant chest injuries were classified according to the AIS_thorax_, and patients were considered as having a concomitant chest injury when the AIS_thorax_ was ≥ 2.

### Classification of TBI

TBIs were classified according to their prehospital Glasgow coma scale (GCS) [16] and after computer tomography scanning. TBI was additionally classified according to the AIS_head_. Patients were only considered as having a concomitant TBI when they had a prehospital GCS ≤ 12 and an AIS_head_ ≥ 2.

### Fracture classification and fracture healing assessment

Only patients with diaphyseal femoral (AO32.A-C) or tibial (AO42.A-C) fractures according to the AO (Arbeitsgemeinschaft für Osteosynthesefragen) classification system were included in the analysis. It was registered if these fractures were open (grade I or II according to Gustilo and Anderson) or closed.

Radiological imaging was reviewed and evaluated by two independent observers (HA and PK), who were blinded to concomitant injuries. A fracture was considered to be consolidated when both observers determined that three out of four cortices were bridged by a callus. Further, callus formation was quantified according to the fracture healing response described by Spencer [17].

### Outcome and complications

In addition to our primary outcome parameter of non-union, further neurological, cardiopulmonary, vascular, urinary tract, orthopaedic and systemic complications were registered.

### Statistical methods

Data were analysed using SPSS (version 25; IBM Inc., Somers, NY, USA). Incidences are presented with counts and percentages, while continuous values are presented as mean ± standard deviation. Differences between the groups were evaluated with Mann–Whitney’s *U* test for continuous data, and Pearson’s *χ*^2^ test was used for categorical values. The nonparametric Spearman’s rank test was used for statistical correlation. Multivariate logistic regression analysis was performed with non-union as the dependent variable to adjust for confounding variables. The results were reported as odds ratio with 95% confidence intervals (95% CI). In general, a two-sided *p*  < 0.05 was considered to be statistically significant.

## Results

### Demographic data

A total of 136 and 68 patients were treated at the University Hospital RWTH Aachen (Germany) and the Maastricht University Medical Centre (The Netherlands), respectively. Of these, 100 patients (49.0%) had femoral fractures and 104 (51.0%) had tibial fractures. Overall, 25 (12.3%) patients had a concomitant chest injury and 27 (13.2%) had a concomitant TBI. A total of 98 patients (48.0%) underwent ETC, and 106 (52.0%) underwent DCO. Conversion to definitive osteosynthesis was performed 6.2 ± 5.7 days after trauma (Table 1).

**Table 1:** Clinical and radiological parameters

	Control group (*n* = 180)	NU group (*n* = 24)	*p* value
Clinical parameters			
General			
Age (years)	35.5 ± 15.5	39.7 ± 15.1	0.167
Gender (female to male ratio)	0.42	0.33	0.652
General health status			
ASA classification system	1.2 ± 0.7	1.3 ± 0.7	0.489
CCI	0.6 ± 1.3	0.8 ± 1.2	0.334
Injury severity			
ISS	11.0 ± 8.8	10.7 ± 7.5	0.869
GCS	14.2 ± 2.4	14.4 ± 2.3	0.622
AIS head	0.4 ± 0.9	0.3 ± 1.0	0.753
AIS thorax	0.4 ± 1.0	0.3 ± 1.0	0.735
Concomitant injuries	54.2%	50.0%	0.699
AO classification	A 55.3% B 36.3% C 8.4%	A 45.8% B 37.5% C 16.7%	0.385
Open/closed fracture	Closed 80.6% Open 19.4%	Closed 66.7% Open 33.3%	0.117
Clinical course			
Duration of hospital stay (days)	17.1 ± 17.6	19.8 ± 14.0	0.535
In-hospital complications**	23.3%	20.0%	0.785
Period trauma: first operative treatment (days)	0.4 ± 1.1	0.6 ± 1.9	0.506
Period trauma-definitive osteosynthesis (days)	3.9 ± 5.3	11.5 ± 38.2	0.012*
Period trauma-consolidation (days)	326.5 ± 278.3	–	
Period definitive osteosynthesis-discharge	15.9 ± 17.4	17.5 ± 14.0	0.663
Period definitive osteosynthesis-consolidation (days)	322.2 ± 277.5	–	
Period trauma-discharge	17.7 ± 17.6	19.8 ± 14.0	0.574
Total number of operations performed	1.5 ± 0.5	2.1 ± 0.8	< 0.001*
ETC vs. DCO	ETC 51.1% DCO 48.9%	ETC 25.0% DCO 75.0%	0.016*
Radiological parameter			
Fracture healing response	1.5 ± 0.3	0.4 ± 0.7	< 0.001*
Consolidation (3 out of 4 cortices)	100%	0%	

*Statistical significance, *p* < 0.05

**Any secondary neurological, cardiopulmonary, vascular, urinary tract, orthopaedic and other complications were registered

### General health status, injury severity and clinical course

The general health status and the injury severity, distribution and characteristics did not significantly differ between the two study groups. Over the clinical course, significant differences for the time period until definitive fracture stabilization (*p* = 0.012), the total number of operations performed before consolidation (*p* < 0.001) and the ratio of ETC to DCO (*p* = 0.016) were observed between the control and NU groups (Table 3).

### Nonparametric correlation analysis referring to non-union

Non-union was diagnosed in 11.8% (*n* = 24) of our patient population. Nonparametric correlation analysis showed a correlation between the fracture healing response and non-union development (*r* = − 0.424, *p* < 0.001). Also, the CCI was correlated with the incidence of non-union (*r* = 0.148, *p* = 0.034). For TBI and chest trauma, no correlation was found (Table 4).

### Multivariate regression analysis referring to non-union

The multivariate regression analysis referring to non-union showed that only the total number of operations before consolidation was an independent risk factor for non-union development (OR = 6.416; *p* < 0.001; Table 2).

**Table 2:** Multivariate regression analysis referring to non-union analysing age, gender, ASA, CCI, ISS, GCS, AIS_head_, AIS_thorax_, concomitant injuries, AO classification, open/closed fracture, period between trauma and definitive osteosynthesis and the total number of operations before consolidation as potential predictors (Nagelkerke: *R*^2^ = 0.294)

Predictor	Regression coefficient	Odds ratio (OR)	95% confidence interval (95%-CI)	*p* value
Patient-specific				
Age (years)	0.039	1.040	0.995–1.086	0.083
Gender (male)	0.203	1.225	0.358–4.186	0.747
ASA	− 0.070	0.933	0.404–2.152	0.870
CCI	0.017	1.017	0.616–1.679	0.947
Injury-specific				
ISS	− 0.022	0.978	0.895–1.069	0.624
GCS	0.513	1.670	0.638–4.369	0.296
AIS_head_	0.579	1.784	0.462–6.897	0.401
AIS_thorax_	− 0.066	0.936	0.489–1.792	0.842
Concomitant injuries	− 0.236	0.790	0.235–2.661	0.704
AO classification	0.630	1.878	0.355–9.954	0.459
Open/closed fracture	0.651	1.917	0.571–6.440	0.292
Treatment-specific				
Period trauma-definitive osteosynthesis (days)	0.042	1.043	0.995–1.094	0.077
Total number of operations before consolidation	1.859	6.416	2.434–16.910	< 0.001*
Constant	− 14.309			0.058

*Statistical significance, *p* < 0.05

Most patients underwent one or two operations and only seven patients underwent more than two operations, which were performed due to hardware failure (*n* = 3) or infection (*n* = 4) and not because of disturbed healing. Therefore, we focused on the patients who underwent either one or two operations. Of these patients, 96 underwent ETC and 88 of the 101 patients who had two operations (87.1%) underwent DCO. Comparing these two subpopulations using non-parametric correlation, DCO strategy is more frequently applied with younger patients (*r* = − 0.277, *p* < 0.001) and in male patients (*r* = − 0.208, *p* = 0.005). Referring to injury-specific aspects, no correlation is found towards open fractures (*r* = − 0.017, *p* = 0.815). Furthermore, patients with a poorer general health status (ASA: *r* = 0.162, *p* = 0.028) as well as patients with a more severe injury pattern (ISS: *r* = 0.471, *p* < 0.001; AIS_head_: *r* = 0.282, *p* < 0.001; AIS_thorax_:* r *= 0.308, *p* < 0.001; AIS_extremity_: *r* = 0.236, *p* = 0.001; concomitant injuries: *r* = 0.449, *p* < 0.001; complications: *r* = 0.314, *p* < 0.001) were more likely to undergo DCO treatment. As described before, correlation between DCO treatment and the development of non-unions was found (*r* = 0.161, *p* = 0.029) (Table 3).

**Table 3:** Non-parametric correlation analysis of DCO treatment

Parameter	Correlation coefficient (*r*)	*p* value
Age	− 0.277	< 0.001**
Gender	− 0.208	0.005**
ASA	0.162	0.028*
ISS	0.471	< 0.001**
AIS head	0.282	< 0.001**
AIS thorax	0.308	< 0.001**
Concomitant injuries	0.449	< 0.001**
Complications	0.314	< 0.001**
AIS extremity	0.236	0.001**
Open/closed fractures	− 0.017	0.815
Duration of hospital stay (days)	0.729	< 0.001**
Non-union	0.161	0.029*

*Statistical significance *p* < 0.05

**Statistical significance *p* < 0.01

In the multivariate regression analysis referring to non-union in DCO vs. ETC, DCO represented an independent risk factor for non-union development with an odds ratio of 7.878 (*p* = 0.005; Table 4).

**Table 4:** Multivariate regression analysis referring to non-union analysing age, gender, ASA, CCI, ISS, GCS, AIS_head_, AIS_thorax_, concomitant injuries, AO classification, open/closed fracture, period between trauma and definitive osteosynthesis and DCO vs. ETC as potential predictors (Nagelkerke: *R*^2^ = 0.215)

Predictor	Regression coefficient	Odds ratio (OR)	95% confidence interval (95%-CI)	*p* value
Patient-specific				
Age (years)	0.036	1.037	0.990–1.086	0.123
Gender (male)	0.406	1.501	0.424–5.313	0.529
ASA	− 0.183	0.833	0.348–1.993	0.681
CCI	− 0.007	0.993	0.568–1.736	0.980
Injury-specific				
ISS	− 0.020	0.980	0.900–1.067	0.646
GCS	0.467	1.596	0.640–3.977	0.316
AIS_head_	0.500	1.648	0.446–6.094	0.454
AIS_thorax_	− 0.069	0.934	0.493–1.769	0.833
Concomitant injuries	− 0.374	0.688	0.185–2.565	0.578
AO -classification	0.680	1.974	0.368–10.583	0.427
Open/closed fracture	0.714	2.042	0.577–7.219	0.268
Treatment-specific				
Period trauma-definitive osteosynthesis (days)	0.039	1.040	0.994–1.088	0.088
DCO vs. ETC	2.064	7.878	1.889–32.860	0.005*
Constant	− 13.780			0.056

*Statistical significance, *p* < 0.05

## Discussion

Non-unions of long bone fractures represent a challenging problem in trauma patients. Patient-, injury- and treatment-specific factors have been previously described to influence the occurrence of non-unions. Independent from the already well-known risk factors for non-union development, we aimed to focus on the impact of fracture stabilisation strategy, chest injury and TBI on the occurrence of non-unions in diaphyseal long bone fractures. Our main results can be summarized as follows:

1. The DCO fracture stabilisation strategy represents an independent risk factor for the development of non-unions in long bone fractures.
2. Chest injury and TBI were not identified as influencing factors for non-union development in diaphyseal long bone fractures.

Although DCO treatment is well accepted to be beneficial in certain subgroups of trauma patients, we found that this treatment strategy is associated with a higher risk of non-union. Our findings were in accordance to those reported in the previous study by Rixen et al. [18]. In particular, the timing of conversion from external fixation to definitive stabilization has been suggested as an indispensable factor for non-union development [19, 20]. In this context, late conversion (> 10 days after the initial treatment) has been associated with an increase in fracture-associated complications, such as non-union [21]. Therefore, it is of utmost importance to plan definitive surgery meticulously. In this context, Pape and Pfeifer revitalized the discussion on the DCO treatment strategy by introducing the concept of safe definitive surgery (SDS). In this concept, the time point of definitive fracture stabilization is based on a regular re-evaluation and assessment of the patient’s physiological condition and not on a suggested time point like in the DCO concept (e.g. not before day 5). The SDS concept; therefore, might lead to a dynamic combination of the advantages of both the DCO and ETC treatment strategy [22]. Our findings support the philosophy of this approach. The decision for DCO treatment strategy in patients with more severe injury and with poorer general health status, as demonstrated in our study, forms a gold standard nowadays. However, according to the SDS concept and taking our study results into consideration, approaches to identify patients who could potentially benefit from DCO should be improved to avoid the increased risk of non-union development.

To assess the relevance of concomitant injuries, we focused on chest injuries and TBI. However, both entities did not significantly influence the development of non-unions. However, it has to be noticed that the overall ISS of our study population was relatively low and the occurrence of chest or brain injuries in these patients is relatively infrequent, which made it especially difficult to demonstrate independent effects of concomitant injuries on fracture healing rates.

In contrast to our study, Recknagel et al. [23] suggest that chest trauma has a negative effect, particularly on the late phases of bone regeneration and fracture healing. A chest trauma-associated hypoxaemia-induced enhancement of local and systemic inflammation has been suggested as a potential pathomechanism by Kemmler et al. [24]. The differences between the results of our study and these experimental studies might be explained with different aspects. First, data obtained in animal experiments under standardized conditions might not be point-to-point transferable to the clinical situation with different confounding factors. Second, it has been postulated that the strategy for fracture fixation is an even more important factor for fracture healing than concomitant injuries [23]. This would be in line with our results and might explain why we did not observe an impact of chest trauma on the incidence of non-unions.

TBI did not have a significant influence on fracture healing. Therefore, the findings of this study are in contrast to the findings of the majority of studies that TBI has a positive influence on bone regeneration [11]. In this context, a retrospective study [8] found shorter healing time and increased callus dimensions in patients with concomitant TBI. In contrast to our study, they excluded patients treated according to the DCO principle. As fracture fixation represented an independent risk factor for disturbed fracture healing in our study, this might be one explanation for the different results of the studies. This assumption would also support the finding of the aforementioned experimental study that fracture fixation has more impact on fracture healing than concomitant injuries [23]. Another clinical study demonstrated shorter healing times, greater callus volumes and higher fracture healing rates in patients with concomitant TBI [25]. In contrast to our study, they included all long bones fractures (including humeral and fibular fractures) treated either with intramedullary nailing or plate osteosynthesis. Furthermore, they included only patients with severe TBI (GCS < 8). These differences are likely to contribute to the differences between that study and our present study.

### Strength and limitations

A strength of our study design is that by strict inclusion and exclusion criteria, we were able to analyse a specific patient cohort with smaller parameter variance and better comparability, in which we could focus on the influence of chest injury, TBI and fracture stabilisation strategy on non-union development by eliminating other possible confounding factors as much as possible.

One limitation of this study is its retrospective design. Second, a large number of patients (114) were lost to follow-up. Some of those patients may have had complications from the treatment and went for care elsewhere. On the other hand, patients with a straightforward healing process may have disengaged from the follow-up because they did not think it was essential. These phenomena could lead to possible selection bias.

Third, the treatment evaluated was limited to reamed intramedullary nailing and in consequence we cannot assess the influence of the studied parameter on non-unions in diaphyseal long bone fractures following other treatment strategies. However, reamed intramedullary nailing is an established technique, and is the preferred therapy for long bone shaft fractures of the lower extremities in adults [5].

## Conclusion

Our results demonstrated that fracture stabilisation strategy is a far more powerful factor than concomitant injuries influencing non-union development in long bone fractures. Based on our finding that DCO stabilisation strategy is an eminent predictor for non-union development, it is of utmost importance in the clinical situation to critically review both: the indication for DCO and the time period until conversion to definitive treatment to minimize the risk of disturbed fracture healing. Our study further counterweights the rising evidence of concomitant chest injury predisposing and concomitant TBI protecting for non-unions in the specific situation of diaphyseal long bone fractures of the lower extremities. These findings could contribute to the improvement of the treatment principles and to the reduction of the treatment costs of non-union and its sequelae. Furthermore, reliably predicting the risk of non-union in certain fractures at the time of initial treatment would be a great advantage and could possibly modify treatment management.

## Abbreviations

AIS: Abbreviated injury scale
AO: Arbeitsgemeinschaft für osteosynthesefragen
ASA: American College of Anaesthesiologists
ATLS^®^: Advanced Trauma Life Support^®^
CCI: Charlson comorbidity index
DCO: Damage control orthopaedics
ETC: Early total care
FHR: Fracture healing response
GCS: Glasgow coma scale
ISS: Injury severity score
OR: Odds ratio
SPSS: Statistical package for the social sciences
TBI: Traumatic brain injury
